# Awareness of asthma and its management in primary school teachers in Baghdad, Iraq

**DOI:** 10.12688/f1000research.73495.1

**Published:** 2022-03-30

**Authors:** Muhannad R. M. Salih, Arwa Y. Abd, Hayder Adnan Fawzi

**Affiliations:** 1 Department of Pharmacy, Al Rasheed University College, Baghdad, Iraq

**Keywords:** primary school, teachers, knowledge, confidence, asthma, management, children

## Abstract

**Background:** Asthma is a major global health issue characterized by chronic airway inflammation. It is linked to a high disease burden and disproportionately high healthcare utilization in severe, uncontrolled cases compared to non-severe asthma. We aimed to conduct this survey among primary school teachers in Baghdad, Iraq, to assess their level of knowledge about asthma and confidence in managing school children with asthma.

**Methods: **This was a prospective cross-sectional study conducted in the Karkh and Rusafa areas of Baghdad. The study adopted a questionnaire for assessing the asthma knowledge and confidence scores regarding the management of asthmatic children. This questionnaire contained 29-multiple true-false questions based on different aspects, including facts about asthma and the management of asthma by teachers.

**Results: **The questionnaire for testing teachers’ knowledge of asthma and confidence scores regarding the management of asthmatic children was distributed to 150 teachers. 103 (68%) teachers completed the questionnaire fully. Approximately 71% of teachers answered the question ‘What are the three main symptoms of asthma?’ correctly i.e., answering either one symptom (35.9%) or two symptoms (35.9%) correctly. A relatively smaller number of teachers (16.5%) mentioned all three symptoms correctly. Across the 29-multiple true-false questions, more than 75% of teachers answered 11 questions correctly, 50-74% of teachers answered the rest of eight questions appropriately, and <50% of teachers answered the remaining 10 questions properly. The statistical evaluation indicated that the mean total knowledge score about asthma for all the teachers was 20.27 ± 2.97 and the mean total confidence score regarding the management of asthmatic children was 72.44 ± 13.61.

**Conclusions: **This study suggests that teachers from the schools in Karkh and Rusafa areas of Baghdad appear to be self-confident in their ability and knowledge to help and manage children with asthma.

## Introduction

Asthma is one of the most common chronic diseases in children globally. ^1^ During the last 40 years, there has been a significant rise in the prevalence, morbidity, and mortality related to asthma in children worldwide. ^2^ As per the World Health Organization (WHO), there are more than 339 million asthmatic cases globally. ^3^ The global death rate in asthmatic children is 0 to 0.7 per 100,000 people and asthma is listed in the top 20 conditions globally for disability-adjusted life years in children. ^2^ The incidence of asthma in Baghdad, Iraq, is closely 22%. ^4^ In a cross-sectional study carried out between October 2000 and June 2002 conducted by Al-Thamiri D *et al.,* in Baghdad, Iraq, asthma was diagnosed in approximately 82% of primary-school children who had experienced wheezing and difficulty breathing in the last 12 months at the time of the administration of the questionnaire. ^5^

It is an important causative factor for school absenteeism in children and decreased involvement of children in school activities. ^6^^,^^7^ Meng YY *et al.,* ^6^ reported that students with frequent asthmatic symptoms and those who were on asthma medications had increased likelihood of missing school. As per a review by Elif Isik RN *et al.,* ^7^ uncontrollable asthma leads to a significant rise in visits to an emergency room, hospitalization, and school absenteeism that eventually contributes to emotional and financial problems for parents, and decreased school performance in children. School activities including extracurricular activities are restricted in asthmatic children, and uncontrollable asthma affects social interactions and self-confidence in children.

Most of the schools in Baghdad do not have full-time nurses to manage children with asthma. These circumstances lead to imparting the responsibility of managing school children with asthma onto the non-medical staff or schoolteachers. Various studies have reported that schoolteachers have inadequate awareness about asthma and the management of asthma among school children. ^8^^–^^11^ Therefore, it is recommended to train schoolteachers on specific aspects of asthma and its management for school children. The study aims to assess teachers’ current knowledge about asthma and to infer the need for teacher training around asthma and asthma management in school children. Therefore, we aimed to conduct this survey among primary school teachers in Baghdad, Iraq, to assess their level of knowledge about asthma and confidence in managing school children with asthma.

## Methods

### Ethics approval

The study has been approved by the Ethical Committee of the Ministry of Higher Education and Scientific Research Al-Rasheed University College, Department of Pharmacy (approval number 121) on the 3 ^rd^ September, 2019. There was no established ethical committee for controlling such research purposes at the governmental authority in charge of primary schools in Iraq.

### Informed consent

Written informed consent was obtained from all the participants. Oral consent was taken from the principal of each school before distributing the questionnaire to the teachers (since the principal of each school did not participate in the actual study, and only the ethical committee was responsible for approving the protocol, the approval of the principal was a courtesy from the research team to inform the principal about the study).

***Participant recruitment***

This was a prospective cross-sectional study. A sample of eight primary schools in both the parts of Baghdad (Karkh and Rusafa) was targeted during this investigation. All the teachers (excluding support staff, i.e., non-teaching staff) were invited to participate in the survey. The Ministry of Education provided us with a list of the primary schools in Baghdad, including urban and rural (all school must teach primary level students), private and public schools, and education level of the teachers. We randomly selected schools based on this list (we used the excel program to generate random model for selecting the school). The teachers received letters; the letters outlined the study’s purpose as well as the instructions for filling out the questionnaire that was attached. Before the teachers administered the questionnaire we went to the schools and explained the questions and directives in further detail. The selected schools must have students with asthma, the schools must be coeducational (both girls and boys), and the teachers must have at least five years’ experience in the education sector to be included in the study.

The study was conducted during the period of 1 ^st^ October–30 ^th^ November 2019. The aim was the assessment of asthma knowledge of schoolteachers and their confidence level in the management of children with asthma.

### Study design

The study adopted the amended Newcastle asthma knowledge questionnaire from the Al-Motlaq and Sellick (2013) ^20^ study, who amended the questionnaire developed by Fitzclarence and Henry (1990) *,* from the University of Newcastle, New South Wales, Australia. ^12^ This amendment changed the format of the questionnaire to true-false and replaces four of the questions; in total the questionnaire contained 29 multiple true-false questions based on different aspects, including facts about asthma and the management of asthma (see examples one and two below), and one open-ended question on the three symptoms of asthma resulting in 30 questions in total (20-22).

***Translation and cross-cultural adaptation***

Al-Motlaq and Sellick’s score was translated and cross-culturally adapted in accordance with the ‘Guidelines for the Process of Cross-Cultural Adaptation of Self-Report Measures’. ^24^ Two clinical pharmacists (Muhannad Salih and Arwa Abd) who are native Arabic speakers with outstanding English language skills translated the scale into Arabic. The original short form of the scale, as well as the two translations, were evaluated and discussed with a third clinical pharmacist (Hayder Fawzi) in order to fix any conceptual flaws or conflicts and create one Arabic version of the scale. The scale was then re-translated into English by two native English speakers with good Arabic language skills who were unaware of the original version of the questionnaire or the study’s goal.

***Interpretation of the questionnaire***

Two of the 29 true-false questions regarding ‘knowledge’ of asthma are set out as follows in the examples: 1. ‘More than one in 10 children will have asthma at some time during their childhood’, and 2. ‘Children with frequent asthma should have preventive drugs’. The open-ended question on three symptoms of asthma is as follows: ‘What are the three main symptoms of asthma?’.

Each question received a score ‘1’ if a correct answer is given for each of the 29 true/false questions, and the final open-ended question received a score of ‘1’ for each correctly identified symptom of asthma with a maximum of three points. Incorrect answers were given a score of ‘0’ for all 30 questions. This means the range of scores on ‘knowledge’ is 0-32 (0=no correct responses or symptoms identified; 32=all correct responses and three correct symptoms identified in the open-ended final question). The asthma knowledge questionnaire has been proven reliable in the previous studies prior to minor edits. ^12^^–^^15^ Total asthma knowledge score was estimated based on the accurate responses given to each question by teachers with a maximum test score of 32, additionally we divided the answer into three groups based on percentage of answered correctly: more than 75%, between 50–74%, and less than 50%. ^21^

A questionnaire for confidence scores previously developed by Al-Motlaq and Sellick (2013) ^20^ was also used in this study. The confidence scores of teachers regarding the management of asthmatic children were assessed by a questionnaire ^22^ comprising nine questions as cited in Table 2, for example, a couple of questions are as follows: 1. ‘Keeping asthma from getting worse when the student starts to wheeze or cough’ 2. ‘Giving the appropriate medications to the student during an asthma attack’.

Next, the participating teachers were questioned to rate their confidence level on each element/question by putting an ‘X’ spot on a visual analogue scale of 10 centimetres that ranged from one (means not confident at all) to 10 (means fully confident). ^20^^,^^23^

***Data collection and follow-up***

**Figure 1.**
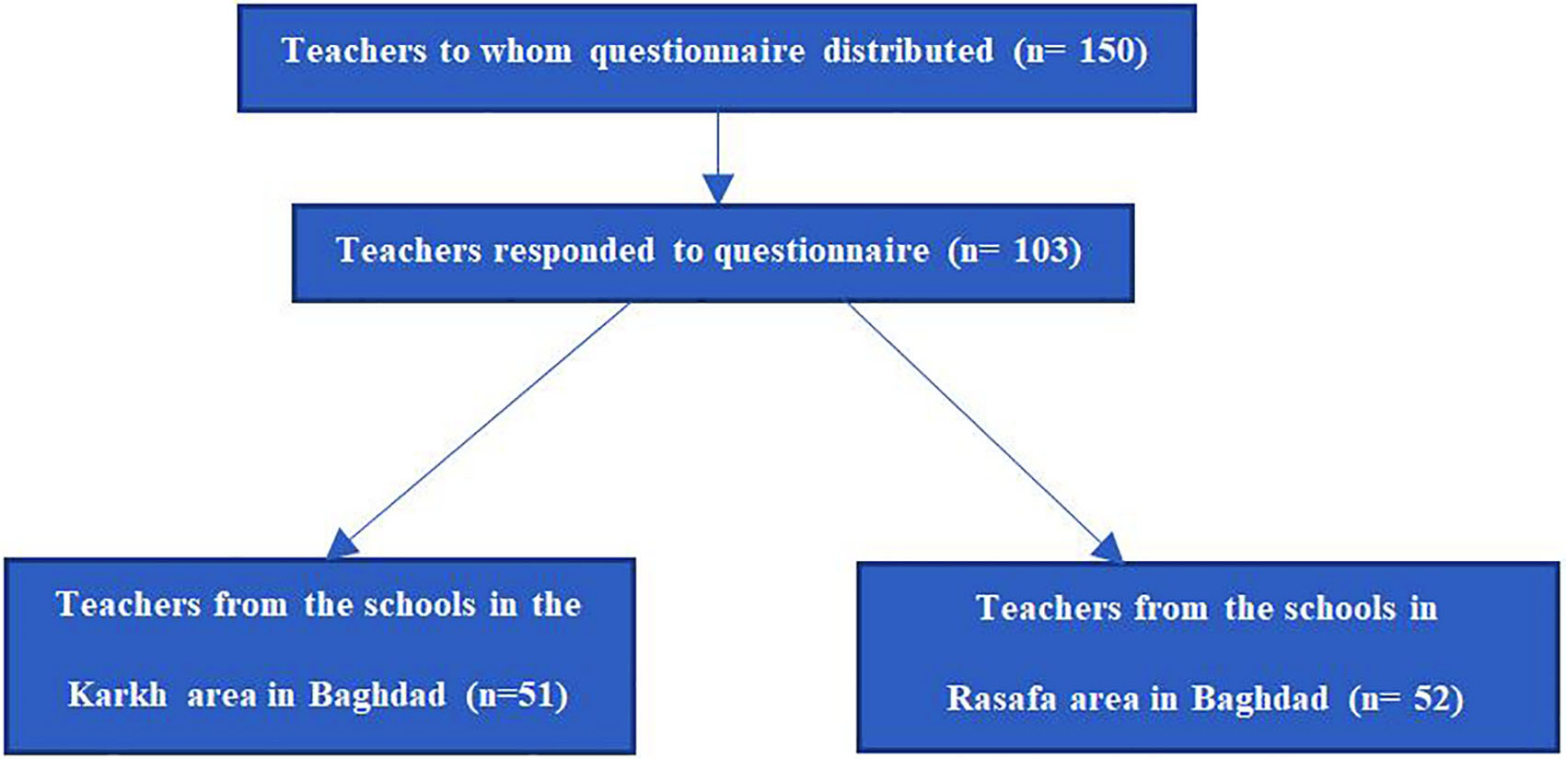
Summary of the number of participants involved in the study of statistical analysis from invitation, responses, and geographical region of Baghdad.

As shown in Figure 1, teachers from eight schools participated in the study. The questionnaire was distributed to 150 teachers in total, the distribution and collection of the questionnaire was done in person by the investigators. 103 teachers (68%) completed the questionnaire in full, the questionnaires were distributed by the authors of this study by direct personal interview to ensure complete understanding of the content of the questionnaire by the teachers. The data were later entered to excel sheets to be sorted later, if there was any missing data the participant was excluded from the final analysis.

After the data collection for this survey, the statistical analysis was done by using IBM SPSS Statistics (IBM Corp. Version 25.0. Armonk, N.Y., USA). The statistics helped to demonstrate the demographics. The categorical variables were denoted with percentages and frequencies. The continuous variables were denoted with the mean ± standard deviation, independent t-test and one way ANOVA were used in this study.

## Results

The socio-demographic characteristics of teachers have been demonstrated in Table 1. Teachers from both private (≈58%) and public (≈42%) schools with ≈50% of schools from both Karkh and Rusafa areas in Baghdad participating in the study (in public school the students fees are paid by the government, while private school the fees are paid by the parents). Participants were primarily female (83%), and reported having a diploma, a bachelor’s, or a master’s degree. A small percentage had diagnosed asthma (10.7%) or a family history of asthma (22.3%). A majority of respondents were over the age of 30 (65%).

**Table 1.** Socio-demographic characteristics of teachers [Total (N)=103].

Group		Frequency (N)	Percent (%)
School type	Private	60	58.3
	Public	43	41.7
Place in Baghdad	Karkh	51	49.5
	Rasafa	52	50.5
Sex	Male	18	17.5
	Female	85	82.5
Asthma diagnosis	Yes	11	10.7
	No	92	89.3
Academic achievement	Diploma	40	38.8
	Bachelor of Arts	56	54.4
	Master’s degree	7	6.8
Family history of asthma	Yes	23	22.3
	No	80	77.7
Age	<30	36	35.0
	30–39	36	35.0
	40–49	21	20.4
	≥50	10	9.7

Teachers’ total knowledge score and the percentage of accurate answers on the asthma knowledge questionnaire are shown in Table 2. 71% of teachers answered the question ‘What are the three main symptoms of asthma?’ with either one symptom (35.9%) or two symptoms (35.9%) identified correctly. A relatively smaller number of teachers (16.5%) mentioned all the three symptoms correctly.

**Table 2.** Teachers’ total knowledge score and the percentage of accurate answers on asthma knowledge (N= 103), Item (“A = Answer”) ^22^.

Items	Answer	Correct (%)
1. What are the three main symptoms of asthma? e.g., Coughing, especially at night, wheezing, and shortness of breath	Non-correct	11.7
	One correct	35.9
	Two correct	35.9
	Three correct	16.5
2. More than one in 10 children will have asthma at some time during their childhood.	(“A=True”)	80.6
3. Children with asthma have abnormally sensitive air passages in their lungs.	(“A=True”)	88.3
4. If one child in a family has asthma then all his/her brothers and sisters are almost certain to have asthma as well.	(“A=False”)	85.4
5. Most children with asthma have an increase in mucus production when they drink cow’s milk.	(“A=False”)	69.9
6. Influenza is a common cause or trigger of an asthma attack.	(“A=True”)	66.0
7. During an attack of asthma, the wheezing may be due to muscles tightening in the wall of the air passages in the lungs.	(“A=True”)	86.4
8. During an attack of asthma, wheezing may occur due to swelling in the lining of the air passage in the lung.	(“A=True”)	65.0
9. Asthma damages the heart.	(“A=False”)	45.6
10. Asthma attacks can be prevented if medications are taken even when there are no symptoms between attacks.	(“A=True”)	59.2
11. Ventolin inhaler is effective during an attack.	(“A=True”)	95.1
12. Antibiotics are an important part of treatment for most children with asthma.	(“A=False”)	35.0
13. Most children with asthma should not eat dairy products.	(“A=False”)	66.0
14. Allergy injections cure asthma.	(“A=False”)	64.1
15. If a person dies from an asthma attack, this usually means that the final attack must have begun so quickly that there was no time to start any treatment.	(“A=False”)	37.9
16. People with asthma usually have nervous problems.	(“A=False”)	31.1
17. Asthma is infectious (i.e., you can catch it from another person).	(“A= False”?)	87.4
18. Inhaled medications for asthma (e.g., Ventolin puffers, rotacaps) have fewer side effects than tablets.	(“A=False”)	68.9
19. Short courses of oral steroids (such as prednisolone) usually cause significant side effects.	(“A=False”)	32.0
20. Some asthma treatments (such as Ventolin) damage the heart.	(“A=False”)	47.6
21. It is better to use inhalers directly, without a holding chamber, so the medication can go more directly to the lungs.	(“A=False”)	48.5
22. When a child has an asthma attack, it’s best to go to the emergency room even if symptoms are mild	(“A=True”)	70.9
23. Children with asthma become addicted to their asthma drugs.	(“A=False”)	34.0
24. Swimming is the only suitable exercise for asthmatics.	(“A=False”)	43.7
25. Parental smoking may make a child’s asthma worse.	(“A=True”)	94.2
26. With appropriate treatment, most children with asthma should lead a normal life with no restrictions on activity.	(“A=True”)	86.4
27. The best way to measure the severity of a child’s asthma is for the doctor to listen to the child’s chest.	(“A=False”)	24.3
28. Asthma is usually more of a problem at night than during the day.	(“A=True”)	75.7
29. Most children with asthma will have stunted growth.	(“A=False”)	86.4
30. Children with frequent asthma should have preventive drugs.	(“A=True”)	94.2

Total knowledge score: Mean=20.27, SD=2.97. Asthma knowledge questionnaire is mostly based on an asthma knowledge questionnaire developed by Fitzclarence *et al.,* *^12^* and modified in Al-Motlaq and Sellick (2013) ^20^ with translation as outlined in the methods section. ^22^

More than 75% of teachers answered the remaining 11 questions correctly (questions: 2, 3, 4, 7, 11, 17, 25, 26, 28, 29, and 3) and a few of those questions are cited as follows: ‘More than one in 10 children will have asthma at some time during their childhood’, ‘children with asthma have abnormally sensitive air passages in their lung’, and ‘if one child in a family has asthma, then all his/her brothers and sisters are almost certain to have asthma as well’, as shown in Table 2.

Moreover, 50–74% of teachers answered remaining eight questions correctly (questions: 5, 6, 8, 10, 13, 14, 18, and 22) and some of those questions are mentioned as follows: ‘Most children with asthma have an increase in mucus production when they drink cow’s milk’, ‘influenza is a common cause or trigger of an asthma attack’, and ‘during an attack of asthma, wheezing may occur due to swelling in the lining of the air passage in the lungs’, etc. as shown in Table 2.

Furthermore, less than 50% teachers answered the remaining 10 questions correctly (questions: 9, 12, 15, 16, 19, 20, 21, 23, 24, and 27) and few of those questions are cited as follows: ‘Asthma damages the heart’, ‘antibiotics are an important part of treatment for most children with asthma’, and ‘if a person dies from an asthma attack, this usually means that the final attack must have begun so quickly that there was no time to start any treatment’, etc. as shown in Table 2.

The mean total knowledge score for all the teachers was 20.27 (SD=2.97). There were no significant differences in asthma-related knowledge scores of teachers based on the types and areas of the schools, age, gender, teachers with an asthma diagnosis, family history of asthma, and academic achievements.

As shown in Table 3, teacher’s confidence scores in managing children with asthma ranged from 55.82 to 86.60 for various parameters and a few of those are cited as follows as examples: **‘**Taking a student on a school camp or excursion’, ‘helping a student to use their inhaler during an asthma attack’, and ‘calming a student when they have the difficulty in breathing’. The teacher’s confidence score was the highest (86.6) for the parameter **‘**helping a student to use their inhaler during an asthma attack’. The overall confidence score in managing children with asthma was 81.75. The mean total confidence score considering all the parameters was 72.44±13.61.

**Table 3.** Teachers’ confidence scores regarding the management of asthmatic children (N=103). ^23^

Item	Mean	SD
1. Taking a student on a school camp or excursion.	55.83	34.14
2. Helping a student to use their inhaler during an asthma attack.	86.60	18.29
3. Calming a student when they have the difficulty in breathing.	78.74	25.69
4. Keeping asthma from getting worse when the student starts to wheeze or cough.	72.72	24.42
5. Handling an asthma attack rather than taking the student to the hospital.	55.73	28.92
6. Giving the appropriate medications to the student during an asthma attack.	71.84	27.47
7. Knowing when the student requires medical assistance.	68.25	27.02
8. Helping a student avoid things they are allergic to.	80.58	24.40
9. Your overall confidence in managing children with asthma.	81.75	22.81

Total confidence score: Mean=72.44, SD=13.61. SD=Standard deviation. Each item for assessing teachers’ confidence scores is mostly based on parameters developed by Fitzclarence and Henry, ^12^ and modified in Al-Motlaq and Sellick (2013). ^20^^,^^22^

Analysis for the association between the total confidence score and sociodemographic characteristics of primary school teachers was performed and results are shown in Table 4. Female teachers showed a significantly higher mean total confidence score ( *p*=0.02) than male teachers. Teachers who had a family history of asthma showed a significantly higher mean total confidence score ( *p*=0.03) than those without such history.

**Table 4.** Association between the total confidence score and sociodemographic characteristics of primary school teachers.

Characteristics		N	Total confidence score (Mean±SD)	*p*-value
School type	Private	60	70.9±13.7	0.19 ^a^
	Public	43	74.5±13.4	
Place in Baghdad	Karkh	51	72.0±14.2	0.74 ^a^
	Rasafa	52	72.9±13.2	
Gender	Male	18	66.2±12.0	**0.02** ^a^
	Female	85	73.8±13.6	
Diagnosed with asthma	Yes	11	77.3±11.3	0.17 ^a^
	No	92	71.9±13.8	
Academic achievement	Diploma	40	70.1±14.0	0.37 ^b^
	BA	56	73.8±13.3	
	Master degree	7	75.1±14.2	
Family history of asthma	Yes	23	77.2±10.7	**0.03** ^a^
	No	80	71.1±14.1	
Age	<30	36	73.2±13.4	**0.003** ^b^
	30-39	36	67.2±13.2	
	40-49	21	74.6±12.6	
	≥50	10	84.2±9.9	

^a^ Independent Sample T-Test.

^b^ ANOVA.

Teachers ≥50 years of age demonstrated a significantly higher mean total confidence score ( *p*=0.003) than other age groups. On the contrary, no significant association was observed between the total knowledge score and sociodemographic characteristics of teachers ( Table 5). Further analysis showed no significant correlation between the total confidence scores and total knowledge scores among primary school teachers (r=0.02, *p*=0.82).

**Table 5.** Association between the total knowledge score and sociodemographic characteristics of primary school teachers.

Characteristics		N	Total knowledge score (Mean±SD)	*p*
School type	Private	60	20.3±2.7	0.82
	Public	43	20.2±3.3	
Place in Baghdad	Karkh	51	20.5±3.3	0.26
	Rasafa	52	20.0±2.7	
Gender	Male	18	19.7±3.3	0.36
	Female	85	20.4±2.9	
Asthma diagnosis	Yes	11	20.2±2.2	0.94
	No	92	20.3±3.1	
Academic achievement	Diploma	40	19.9±3.2	0.63
	BA	56	20.4±2.9	
	Master degree	7	21.1±2.5	
Family history of asthma	Yes	23	20.3±3.2	0.64
	No	80	20.3±2.9	
Age	<30	36	20.3±2.1	0.92
	30-39	36	20.1±3.6	
	40-49	21	20.3±3.1	
	≥50	10	20.9±3.3	

## Discussion

School teachers have a responsibility to take care of children when they are at school, thus they need to be knowledgeable about asthma and to be confident about helping children suffering from asthma. School teachers’ knowledge about asthma may make a difference in the health condition of asthmatic children. Plenty of studies indicate that schoolteachers have limited knowledge about asthma. ^16^^–^^18^

In the present study, the mean total knowledge score [20.27 (SD = 2.97)] about asthma for all the teachers appears to be relatively good in comparison with other studies. Though the teachers’ knowledge score appears to be relatively better than that which was observed in a study conducted by Gibson *et al.* ^19^ (knowledge score = 14.90)’ the study was conducted in New South Wales, Australia and included 1,104 teachers and 4,161 students in 1995. But it was less than that (26.3) observed in a study conducted by Mohammad Al-Motlaq *et al.,* ^20^ in Australia. Thus, it indicates key gaps in the knowledge about asthma in the schoolteachers involved in the present study. ^19^^,^^20^

The current study indicates that teachers’ knowledge scores about asthma are not related to different variables viz. types and areas of the schools, age, gender, teachers with an asthma diagnosis, family history of asthma, and academic achievements. It indicates that these variables did not play a role as confounding variables.

In the current study, the overall confidence score was 81.75 in managing children with asthma. 65% of teachers were in the age group of above 30 years of age. Their maturity levels and experience may have contributed to a higher confidence score in the management of children with asthma. A finding from the current study also demonstrates a significantly higher mean total confidence score in teachers who had a family history of asthma, which is similar to that observed in the study conducted by Al-Motlaq *et al.* ^20^ However, unlike the study by Al-Motlaq *et al.,* female teachers showed a significantly higher mean total confidence score than male teachers in the present study. ^20^ Thus, there could be some other factors that may have contributed to a significantly higher mean total confidence score in female teachers.

The mean total confidence score in managing children with asthma was 72.44 (SD 13.61) in the present study appears to be slightly greater than that observed in a study conducted by Mohammad Al-Motlaq *et al.,* in the Gippsland region of Victoria in Australia. ^20^ Though the mean confidence score with an item ‘your overall confidence in managing children with asthma’ was 81.75 (SD 22.81), an approximate score of ‘55’ with other parameters must have contributed in causing the mean total confidence score in the current study. Responses to these lower score parameters suggest areas for further improvement in the total confidence score of schoolteachers through education and training sessions.

It is encouraging to know that the knowledge related to asthma and confidence of teachers in the management of children with asthma were relatively satisfactory in the present study considering the results from other studies. ^19^^,^^20^ However, training should be recommended to improve teachers’ knowledge score and such training may also help to take teachers’ confidence in managing children with asthma to the next level.

The limitations of this study are as mentioned previously; the study adopted an asthma knowledge questionnaire amended by Al-Motlaq and Sellick (2013) and was originally developed by Fitzclarence *et al*., ^12^ from the University of Newcastle, New South Wales, Australia with a translation into Arabic as outlined in the methods section. However, the asthma knowledge questionnaire was not pre-tested considering that the original version was validated in the Fitzclarence *et al*., ^12^ study and there were only minor amendments involved. ^21^ However, the amended questionnaire used in this study from Al-Motlaq and Sellick (2013) was not validated in their study. In addition, the sample size for the study was not calculated so 150 teachers were considered based on convenient sampling.

## Conclusions

This study suggests that teachers from schools in Karkh and Rusafa areas of Baghdad appear to be reasonably self-confident in helping to manage children with asthma. However, training may be recommended to improve teachers’ knowledge score and such training may help to take teachers’ confidence in the management of children with asthma to the next level.

## Data availability

### Extended data

Zenodo: The modified Newcastle Asthma Knowledge Questionnaire. https://doi.org/10.5281/zenodo.5837458. ^21^

This project contains the following extended data:

• Arabic translation.pdf. (The amended Newcastle Asthma Knowledge Questionnaire translated into Arabic for this study).
• Mohammad Al-Motlaq.png. (Amended Newcastle Asthma knowledge questionnaire from Al-Motlaq and Sellick (2013) in original English).

Data are available under the terms of the Creative Commons Attribution 4.0 International license (CC-BY 4.0).

Zenodo. Confidence score (Arabic and English version). https://doi.org/10.5281/zenodo.6331672. ^22^

This project contains the following extended data:

• Confidence scores questionnaire – Ar (Arabic questionnaire in PDF and Word format).
• Confidence scores questionnaire – EN (English questionnaire in PDF and Word format).

Data are available under the terms of the Creative Commons Attribution 4.0 International license (CC-BY 4.0).

### Reporting guidelines

Zenodo: SRQR checklist for ‘Awareness of Asthma and Its Management in Primary School Teachers of Baghdad, Iraq’. https://doi.org/10.5281/zenodo.5803507. ^23^

Data are available under the terms of the Creative Commons Attribution 4.0 International license (CC-BY 4.0).
