# Supplementary material for: Awareness of asthma and its management in primary school teachers in Baghdad, Iraq
Source: F1000Res. 2022 Sep 26;11:367. Originally published 2022 Mar 30. [Version 2] doi: 10.12688/f1000research.73495.2 (PMC9218586; doi:10.12688/f1000research.73495.2)
Supplement: Peer review history

# Peer review: Reviewer response for version 1

**DOI:** 10.5256/f1000research.77150.r140322

**Published:** 2022-06-22

**Authors:** Kosisochi Amorha

**Affiliations:** 1 University of Nigeria Nsukka, Nsukka, Nigeria

**GENERAL COMMENTS**

 Asthma is a common chronic disease in children. Children in primary school are usually dependent on their parents or caregivers. However, this responsibility shifts to the teachers when these children are in school. In acute asthma attacks, first responders who are knowledgeable and confident can prevent asthma-related deaths. The authors assessed the level of knowledge and confidence that primary school teachers in Baghdad, Iraq, have in managing school children with asthma. The relevance of this study is further buttressed by the findings of a previous study which reported that more than four-fifths of primary school children in Baghdad were diagnosed of asthma.

 Overall, the article is well-written. The findings of the research add to the body of knowledge. The recommendation of training of primary school teachers in the management of asthma cannot be overemphasized. Despite the knowledge-base of the teachers and their confidence in managing asthma being relatively satisfactory, there is always room for improvement.

**OTHER POINTS**

 Few observations are outlined below:

**Abstract**

- Results: *Note: Do not start a sentence with figures.*
- Arrange keywords in alphabetical order.

**Methods**

- Page 3 (Participant recruitment): …in both parts of Baghdad (Karkh and Rusafa)…
- Page 4 (Data collection and follow-up): Include level of statistical significance, with respect to *p*-value. This is necessary so readers can understand the implications of the p-values in the *Results *section.

**Results**

- Page 5: More than half (71%) of teachers answered… ( *Note: Do not start a sentence with figures*).

**Discussion**

- Page 10: More than three-fifths (65%) of the teachers were in the age group of above 30 years of age. ( *Note: Do not start a sentence with figures*).
- Page 10: … were considered , based on convenience sampling.

Is the work clearly and accurately presented and does it cite the current literature?

Yes

If applicable, is the statistical analysis and its interpretation appropriate?

Yes

Are all the source data underlying the results available to ensure full reproducibility?

Yes

Is the study design appropriate and is the work technically sound?

Yes

Are the conclusions drawn adequately supported by the results?

Yes

Are sufficient details of methods and analysis provided to allow replication by others?

Yes

Reviewer Expertise:

Clinical Pharmacy

I confirm that I have read this submission and believe that I have an appropriate level of expertise to confirm that it is of an acceptable scientific standard.

# Peer review: Reviewer response for version 1

**DOI:** 10.5256/f1000research.77150.r129353

**Published:** 2022-05-04

**Authors:** Ramadan M. Elkalmi

**Affiliations:** 1 Faculty of Medicine, Sebha University, Sebha, Libya

I found it interesting to review this article on "Awareness of asthma and its management in primary school teachers in Baghdad, Iraq"

**Abstract**

*Methods:* the authors need to improve the methods in a way to give clear views about the methods, i.e. some of the methods' issues have been presented in the results, which could affect the scholarly of the manuscript. In general, rearrangement of the abstract, paragraphs is advised.

**Introduction:**

 The approach of leading your reader from the Awareness of Asthma and its management in primary school teachers to the goal of this paper was good.

 However, Construction and rearrangement of the last paragraph are recommended as;

*"Various studies have reported that schoolteachers have inadequate awareness about Asthma and asthma management among school children. ^8–11^ Therefore, it is recommended to train schoolteachers on specific aspects of Asthma and its management for school children. Therefore, we aimed to conduct this survey among primary school teachers in Baghdad, Iraq, to assess their knowledge about Asthma and confidence in managing school children with Asthma. The study aims to assess teachers' current knowledge about Asthma and to infer the need for teacher training around Asthma and asthma management in schoolchildren."*

**Methods:**

 This section is well written, but you still need to arrange the sections to give an overview of the study design clearly, to ease the reader's follow-up and understanding of the steps of the study execution and avoid any ambiguity.

**Discussion:**

 Although the discussion section is well written, the summary of the article was good, and the comparisons were relatively coherent.

*Technically:* There is a clear and significant influence of the study of Mohammad Al-Motlaq on the design and theoretical frame of this study. Adding more references on the same topic at the national or regional level is imperative to give a closer picture of Asthma and its management in Iraq schools.

Is the work clearly and accurately presented and does it cite the current literature?

Yes

If applicable, is the statistical analysis and its interpretation appropriate?

Yes

Are all the source data underlying the results available to ensure full reproducibility?

Yes

Is the study design appropriate and is the work technically sound?

Yes

Are the conclusions drawn adequately supported by the results?

Yes

Are sufficient details of methods and analysis provided to allow replication by others?

Yes

Reviewer Expertise:

Clinical pharmacy practic

I confirm that I have read this submission and believe that I have an appropriate level of expertise to confirm that it is of an acceptable scientific standard.

# Response

**Published:** 2022-05-24

**Authors:** Muhannad R. M. Salih

**Affiliations:** Al Rasheed University College, Iraq

***Dear Reviewer***

***Greetings***

 Thank you for your remarks.

**Regarding the abstract:**

 We have separated the sentence in the results that is best represented in the methods in the abstract.

**Regarding the introduction:**

 We have rearranged the last paragraph as recommended :

 ‎

*"Various studies have reported that schoolteachers have inadequate awareness about Asthma and asthma management among school children.* *^8–11^* *Therefore, it is recommended to train schoolteachers on specific aspects of Asthma and its management for school children. Therefore, we aimed to conduct this survey among primary school teachers in Baghdad, Iraq, to assess their knowledge about Asthma and confidence in managing school children with Asthma. The study aims to assess teachers' current knowledge about Asthma and to infer the need for teacher training around Asthma and asthma management in schoolchildren."*

**Regarding the methods:**

 We rearrange it according to instructions.

**Regarding the discussion:**

 We rearrange it according to instructions.

 Thank you for your notes
